# Engineered Nanomedicine with Alendronic Acid Corona Improves Targeting to Osteosarcoma

**DOI:** 10.1038/srep36707

**Published:** 2016-11-08

**Authors:** Tuyen Duong Thanh Nguyen, Arunkumar Pitchaimani, Santosh Aryal

**Affiliations:** 1 Department of Chemistry, Kansas State University, Manhattan, KS 66506, USA; 2 Nanotechnology Innovation Center of Kansas State (NICKS), Kansas State University, Manhattan, KS 66506, USA; 3 Department of Anatomy and Physiology, Kansas State University, Manhattan, KS 66506, USA

## Abstract

We engineered nanomedicine with the stealth corona made up of densely packed bone seeking ligand, alendronic acid. In a typical nanoconstruct, alendronic acid is conjugated with hydrophilic head moiety of phospholipid that has an ability to self-assemble with hydrophobic polymeric core through its hydrophobic long carbon-chain. Proposed nanomedicine has three distinct compartments namely; poly(l-lactic-co-glycolic acid) polymeric core acting as a drug reservoir and skeleton of the nanoconstruct, phospholipid monolayer covers the core acting as a diffusion barrier, and a densely packed alendronic acid corona acting as a stabilizer and targeting moiety. Thus engineered nanomedicine attain spherical entity with ~90 ± 6 nm having negative zeta potential, −37.7 ± 2 mV, and has an ability to load 7 ± 0.3 wt% of doxorubicin. *In-vitro* bone targeting efficiency of nanomedicine was studied using hydroxyapatite crystals as a bone model, and found significant accumulation of nanoparticle in the crystals. Moreover, cellular internalization studies with mouse osteosarcoma confirm the selectivity of nanomedicine when compared to its internalization in non-targeted mouse melanoma. This nanomedicine shows prolong stability in serum and deliver the drug into the cell exhibiting an IC50 of 3.7 μM. Given the strong interacting property of alendronic acid with bone, the proposed nanomedicine hold promises in delivering drug to bone microenvironment.

According to the American Cancer Society, an estimated 3,300 new cases of primary bone cancer are expected to occur during 2016^1^. Although this number just accounts for 0.2% of new cancer diagnoses, bone is one of the most common sites to depot migrating cancerous cells from distant organs owing to its largest bodily scaffold covering from head to toe around compartmentalized organs. Every year, approximately 80% of breast, lung, and prostate cancer patients ultimately develop bone metastasis, which further entry the disease into an incurable phase^2^,^3^. Since the connection between bone microenvironment and cancerous cells was proposed by Stephen Paget in 1889, this metastatic phenomenon has been extensively studied and widely accepted as “soil and seed” relation in which the unique property of bone microenvironment provides a favorable environment for cancerous cells to develop, survive, and proliferate^4^,^5^. Specifically, once cancerous cell homing to bone marrow, it starts to interfere bone remodeling process by a complex cascade of events including upregulating the expression of receptor activator of nuclear factor κB ligand (RANKL); thereby, activating bone resorption via receptor activator of nuclear factor κB (RANK) on osteoclast to assist its growth and expansion^6^,^7^,^8^. This in turn leads to the bone being broken down without new bone being made i.e.; immoderate production of osteoclasts, or bone being made without breaking down old bones i.e.; excessive production of osteoblasts. With the abnormal acceleration or deceleration in osteoclasts and osteoblasts production, bone releases its mineral, becomes more fragile, porous, and consequently leads to bone fracture.

Despite intensive efforts in the development of a therapeutic agent for cancer occurring at bone, tumor localized in bone still remains as an incurable fatal disease due to either the fast clearance or non-specific binding profile of therapeutic agents. In addition, due to the solid composition and larger surface area of bone, targeting therapeutics to the desired location is the major problem in treating bone cancer. The difficulty of eliminating bone-residing cancer necessitates novel alternative treatment regimens to manipulate the tumor cells, drug resistance, and their microenvironment, with minimal off-target effects.

Among different types of bone targeting ligands, bisphosphonate has been long emerging as a bone-seeking agent owing to its greatly binding affinity with hydroxyapatite - a major mineral component in bone environment. In addition, with the acidic property and hydrophilic nature, bisphosphonate’s permeability through the cellular membrane is insignificant, which in turn makes it more extensively accumulates in skeleton than other organs after the administration^9^,^10^. Once considering bisphosphonate’s distribution within the skeletal system, researches have shown that bisphosphonate accumulates more in bone defect site where high bone turnover is associated^11^,^12^. High bone turnover occurs when the activity of osteoclasts and osteoblasts are uncontrolled or are aggressive. Taking an advantage of these properties at bone lesion sites, the bisphosphonate conjugation can be a promising approach to design targeted chemotherapy for bone cancer treatment. Moreover, the antiresorptive properties of bisphosphonate make it suitable combination candidate with other drugs to treat cancer at bone^13^.

Recently, studies have been focused on utilizing bisphosphonate to construct bone-homing nanomedicine by either conjugating alendronic acid (a member in bisphosphonate class) with polymeric backbone or chemotherapeutic drugs via polyethylene glycol (PEG) linker^14^,^15^,^16^,^17^,^18^. These targeted nanocarriers possess common stealth properties provided by well-hydrated PEG moiety decorated on the surface which could evade nanoparticle from reticuloendothelial system (RES). In 2006, Uludag *et al*. reviewed in an attempt to engineer bone seeking therapeutic agents based on formulating therapeutic agents with bisphosphonates^19^. In this review authors have summarized various classes of bisphosphonate and therapeutic agent conjugates such as: small molecule drugs, protein, and imaging agents capable of targeting bone. In a recent study conducted by Swami *et al*., bortezomid, a proteasome inhibitor, loaded polymeric nanoparticle was proposed, in which the stealth PEG corona was conjugated with alendronic acid to target bone^17^. Despite promising achievements in enhancing antitumor activity on mice bearing tumor models, a number of limitations and challenges related to those systems need to be considered. For examples, the acceleration on using PEG moiety in both pharmaceutical and non-pharmaceutical products have consequently led to anti-PEG antibody development in human body^20^,^21^,^22^,^23^,^24^. A recent finding shows that 22–25% occurrence of anti-PEG in healthy blood donors is most probably due to the greater exposure to PEG containing consumer products^24^. Therefore, a substitute material needs to be developed in order to diminish the excessive usage of PEGylated products. Considering the important role of hydrophilic corona layer on the surface of nanoconstruct in stabilizing nanosystem, we hypothesized that the Alendronic acid moiety with hydrophilic phosphate groups could provide favorable environment for water to form hydration layer around the particle protecting it from opsonization when properly structured in nanoconstruct and further sustain the stability of nanosystem under physiological condition.

To this end, we proposed a targeted nanoparticle (TNP) which is made up of Alendronic acid modified lipid and PLGA polymeric core encapsulating chemotherapeutic drug - Doxorubicin (DOX) to simultaneously offer combinatorial actions including targeting and therapy of bone cancer treatment. Proposed nanostructured construct has three distinct layers (1) PLGA core acting as a skeleton of the nanoparticle and drug reservoir, (2) lipophilic phospholipid layer acting as a middle passivating layer and diffusion barrier for the encapsulated drug, and (3) hydrophilic alendronic acid, an outer corona layer, acting as stabilizer and driving of nanoparticle to its target. This densely packed phospholipid conjugated alendronic acid creates a sufficiently thick hydrated shell and prevents nanoparticle from being disassembled. Therefore, the engineered nanomedicine not only has stealth properties providing by bone mineral targeting moiety but also deliver a large quantitative amount of therapeutic agent which could enhance the effectiveness of treatment.

## Result and Discussion

### Synthesis and characterization of ALE-Lipid conjugate

**Figure 1:**
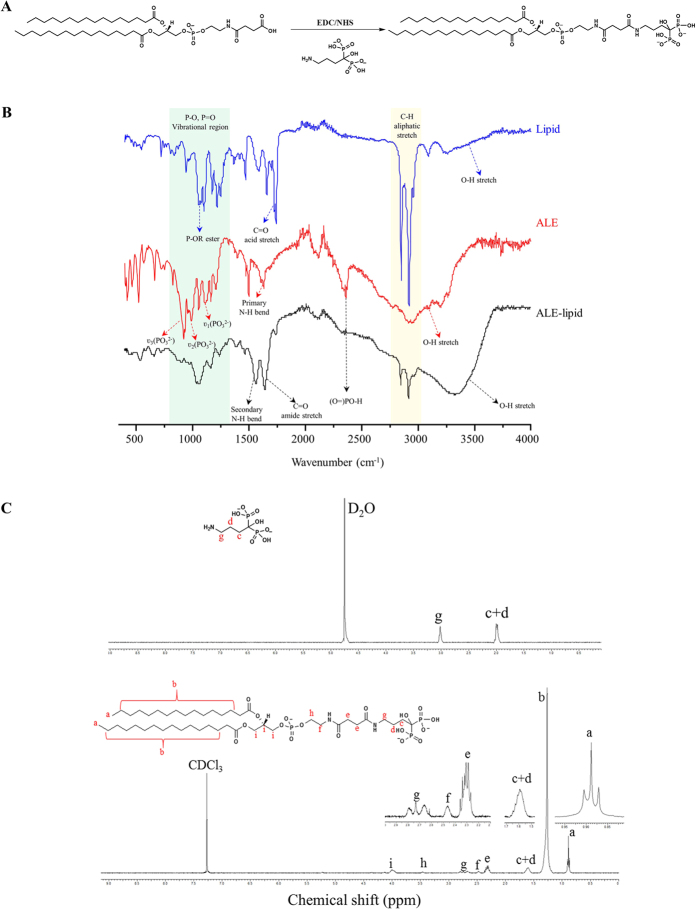
Chemical characterization of synthesized ALE-lipid. (**A**) Coupling reaction scheme. (**B**) FT-IR spectrum showing functional peaks of starting materials (Alendronic acid, Lipid) and product (ALE-Lipid). (**C**) ^1^H-NMR spectra of Alendronic acid in D_2_O and ALE-lipid in CDCl_3_ and its corresponding proton signals.

The synthesis of the lipid conjugate was carried using ethylcarbodiimide hydrochloride/N-hydroxysuccinimide (EDC/NHS) conjugation chemistry as described in Fig. 1A. The chemical structure of synthesized ALE-Lipid conjugate was confirmed by FT-IR and ^1^H-NMR. As shown in Fig. 1B, the FT-IR spectrum of ALE-Lipid (spectrum in black) exhibits all of the characteristic peaks of both unconjugated lipid (spectrum in blue) and alendronic acid (spectrum in red) including strong signal of aliphatic C-H stretch at 2900 cm^−1^ corresponds to lipid backbone and broad O-H stretch at 3500–3100 cm^−1^ belongs to ALE moiety (Fig. 1B). It is notable that after the conjugation, C = O acid stretch of lipid at 1750 cm^−1^ was shifted to 1650 cm^−1^ due to the formation of C = O amide. Most importantly, broad peak ranges at 1300–750 cm^−1^ correspond to phosphate functional groups was broaden in ALE-lipid spectrum which could be attributed to the overlap of phosphate ester (1050 cm^−1^) and phosphonate vibration (985 cm^−1^, 1050 cm^−1^, and 1205 cm^−1^) of lipid and ALE, respectively. In addition, the primary N-H bend (1700 cm^−1^) in ALE spectrum was replaced by secondary N-H bend (1550 cm^−1^) in the spectrum of the conjugated product; thereby confirming the formation of an amide bond. Furthermore, in order to understand the structure of ALE-lipid in detail we conducted the ^1^H-NMR study. In the ^1^H-NMR spectrum (Fig. 1C), Alendronic acid gave a simple spectrum characterized mainly by a triplet at 2.91 ppm corresponding to N-CH_2_ protons and two complicated multiplets arise at 1.9 ppm and 1.88 ppm due to magnetically non-equivalent nature of prochiral CH_2_ protons^25^. In the case of synthesized ALE-Lipid, proton signals that relate to lipid were observed at 1.26 ppm owing to the methylene groups of the long hydrocarbon chain and proton signals of terminal methyl group appear as a triplet at 0.88 ppm. The presence of ALE moiety in the conjugated product was confirmed by a small broad peak appears at 1.6 ppm and triplet locates at 2.73 ppm which were assigned for -CCH_2_CH_2_CH_2_NHCO- and CH_2_NHCO-, respectively. With the formation of ALE-Lipid conjugate these proton signals were upfield shifted from their original chemical shift to 1.8 ppm and 2.91 ppm (in ALE spectrum), respectively, due to the emergence of amide bond and the attachment of lipid, which further verifies the presence of covalently conjugated ALE on the fatty acid backbone.

### Physicochemical properties of nanoparticles

**Figure 2:**
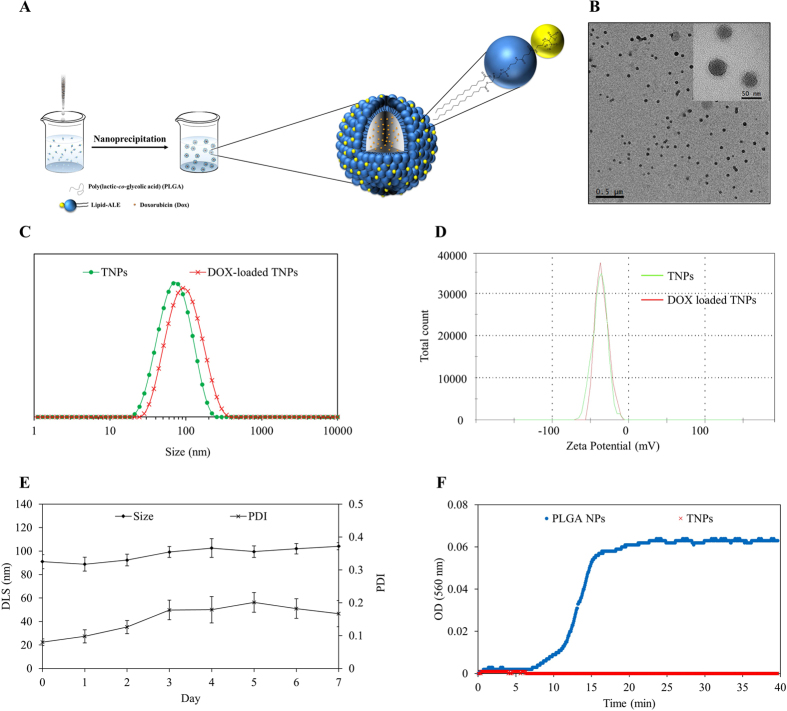
Characterization of TNPs. (**A**) Schematic demonstration of nanofabrication technique and components of TNPs. (**B**) TEM showing morphology and uniformity of TNPs. (**C**) Dynamic light scattering showing hydrodynamic size of TNPs and DOX-loaded TNPs. (**D**) Surface Zeta potential. (**E**) Stability test conducted in ionic condition (PBS, pH 7.4). (**F**) Kinetic stability study in 10% Fetal Bovine Serum (FBS). Values represent mean ± s.d., n = 3.

After successfully attaching ALE with lipid (ALE-lipid), conjugate ALE-lipid was used along with PLGA and DOX for nanofabrication of TNP and DOX-loaded TNP using nanoprecipitation (Fig. 2A). The physicochemical properties and morphological characterization of TNP and DOX-loaded TNP were determined using dynamic light scattering (DLS), surface zeta potential, and transmission electron microscopy (TEM) as shown in Fig. 2B–D respectively. The hydrodynamic size of the TNP showed a diameter of 69 ± 5 nm whereas DLS size of DOX-loaded TNP was found to be 90 ± 6 nm. Importantly, in both cases, the DLS data showed a unimodal distribution with low polydispersity index (PDI) which demonstrates that the TNP and DOX-loaded TNP are highly monodispersed in aqueous solution. The TEM image of TNP further confirmed the uniformity of nanoparticles with the size is around 50 nm. The significant different between hydrodynamic size and dry stage diameter is likely due to well thick hydrating layer in which the densely packed hydrophilic phosphate moiety acting as a corona of the nanoparticle. This hydrophilic corona layer works in a similar way to that of PEG moiety which could make nanoparticle bypass immune system and prolonged the circulation time. By taking this advantage, we not only avoid overusing of PEG moiety but also creating new material that can give similar stealth property.

In addition, the measurement of the TNP and DOX-loaded TNP surface zeta potential revealed a net charge of −37.7 ± 2 mV owing to the presence of negatively charged phosphate moiety on the surface. The similarity in zeta potential value of bare and drug loaded nanoparticles further confirmed the unchanged of surface property of TNP after DOX encapsulation. Taking into account that DOX is the cationic drug, if it is nonspecifically absorbed onto the surface of TNP, the surface charge property of this NPs could change, however, after DOX encapsulation, the surface charge of TNPs remains constant. This observation gives strong evident for the localization of DOX into the core of NPs.

Besides physicochemical properties, the stability of nanoparticles is another essential parameter that needs to be carefully evaluated in order to translate to biological application stage. Therefore, we conducted stability tests in various *in-vitro* physiological conditions including ionic and pooled protein milieu at 37 °C using phosphate buffer saline (PBS) and Fetal Bovine Serum (FBS), respectively. After 7 days of incubation in PBS (pH 7.4), there is no noticeable nanoparticles aggregation was observed demonstrating by unchanged in DLS and PDI indexes (Fig. 2E). In addition, TNPs are found to be highly stable in its colloidal state when expose to serum environment as revealed by constant optical density at 560 nm (Fig. 2F), whereas in the case of bare PLGA NPs, the absorption at 560 nm increase rapidly and reaches to plateau within 15 mins indicating rapid aggregation of PLGA NPs. These kinetic absorption experiments conducted at 560 nm is the measurement of aggregation when NPs aggregated with protein and precipitated to block transparency of the light from the medium^26^. These results implied that the TNPs exhibit robust stability which could enlarge therapeutic window by prolonging circulation time in the bloodstream and enhancing chances of designed nanoparticles to target and accumulate at the bone tumor site. This serum stability further supports our claim that the hydration layer around the NPs due to hydrophilic phosphate moiety is sufficient enough to act as a stealth layer of protection in a manner similar to that of the PEGylated system.

### Calcium binding affinity

**Figure 3:**
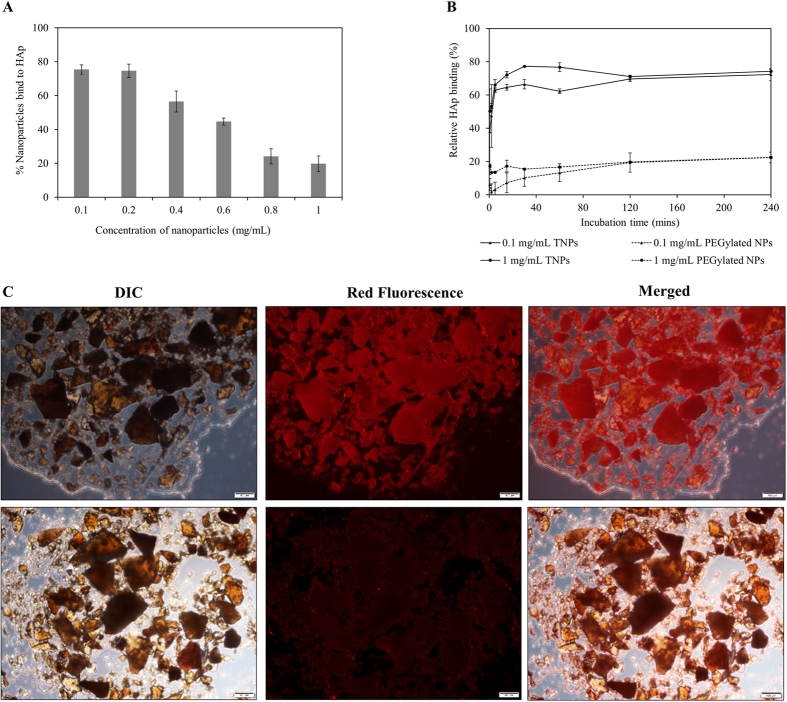
TNP’s interaction with Hydroxyapatite (HAp). (**A**) Quantitative evaluation of HAp binding of NPs with varying concentration of TNPs. (**B**) Time-dependent binding of TNPs with HAp. PEGylated NPs were used as a control. (**C**) Representative fluorescence images of HAp crystal after incubation with RhB-labeled TNPs (upper panel) showing the interaction between HAp and targeted NPs. Lower panel represents non-targeted PEGylated NPs. Values represent mean ± s.d., n = 3.

Since the surface of TNPs was decorated with a bisphosphonate, we next investigated *in-vitro* binding affinity of TNPs with hydroxyapatite (HAp) as a bone-model. Our expectation is that with an increase in NP concentration treated with HAp, the more NPs bound to HAp. However, as the nanoparticle concentration increases, percentage nanoparticle bind to HAp decrease (Fig. 3A). This realized us to optimize the concentration of TNPs that need to be treated with HAp, and the results for optimized concentration is presented in Fig. 3A. With the optimized concentration, we found that nearly 80% of NPs bind to HAp at a concentration of 100 μg/mL, whereas only 20% of NPs were bound at the concentration of 800 μg/mL and 1000 μg/mL. Therefore, we selected minimum and maximum concentration (100 μg/mL and 1000 μg/mL, respectively) to elucidate kinetic binding property of TNPs toward HAp. Along with targeted NPs, PEGylated NPs at the same concentrations was used as a control sample. To our expectation, more than 80% TNPs bound to the surface of HAp within 15 mins of incubation time (Fig. 3B). This trend plateaued on a further increase of incubation time. In contrast, in the case of PEGylated NPs, just 20% of NPs bound to HAp after 2 hours of incubation, at this stage when bounded PEGylated NPs with HAp were centrifuged down and the supernatant was measured spectrophotometrically under fluorescence reader which shows sufficient amount of NPs. This non-specific binding of control NPs could be accounted for carboxylic functional groups presence on the surface of PEGylated NPs. The interaction between HAp and TNPs labelled with RhB was further confirmed by imaging of HAp crystal under fluorescent microscope where the presence of TNP highly enhanced fluorescent signal captured on the surface of incubated HAp crystals, while insignificant fluorescent signal was obtained on HAp samples incubated with PEGylated NPs (Fig. 3C,D, respectively).

### Drug loading and release study

**Figure 4:**
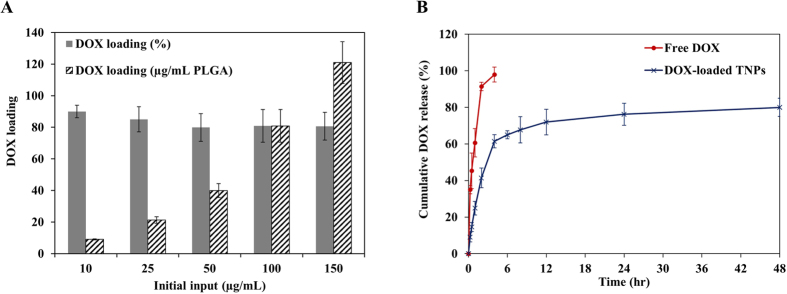
Drug loading and release study. (**A**) Dox loading efficiency and (**B**) *In vitro* doxorubicin release study at pH 7.4. Values represent mean ± s.d., n = 3.

TNP’s drug loading capacity and drug release profile were also evaluated. Figure 4A shows the DOX encapsulation efficiency of TNPs at varying DOX concentrations. There was more than 7 wt% of DOX was successfully encapsulated in 1 mg/mL PLGA. Among these five different formulations, 100 μg/mL initial input of DOX gave the most effective loading efficiency without changing nanoparticle physicochemical property. Thus, this formulation was chosen to use in further experiments.

With the optimized DOX loaded TNPs sample, the *in-vitro* drug release was investigated at pH = 7.4 in phosphate buffer saline (PBS). A cumulative drug release study was performed using 10 kDa molecular cut-off dialysis bags. A control experiment was also performed using an aqueous solution of free DOX placed in the dialysis tubing. As can be seen in Fig. 4B, in the case of free DOX, 100% drug was burst released within the period of 6 h, whereas DOX loaded TNP shows the extension of drug release up to 24 h. This result indicates that DOX loaded TNPs exhibit typical sustained drug release profile of nanomedicine over a 24 h time period at 37 °C (Fig. 4B).

### Cellular uptake study

**Figure 5:**
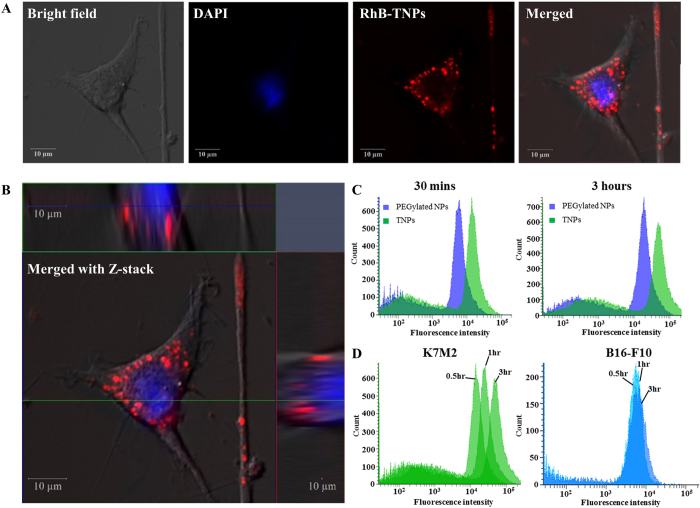
Cellular internalization study. (**A**) Confocal microscopic images of Rhodamine labeled TNPs. (**B**) Z-stack of single K7M2 cell incubated with Rhodamine-labeled targeted nanoparticles showing the intracellular distribution of particles throughout the cytoplasm and perinuclear regions. (**C**) Time-dependent fluorescence-activated cell sorting (FACs) studies showing TNPs internalization pattern into the K7M2 cell. PEGylated NPs without ALE-Lipid were used as a control NPs. (**D**) Selectivity of TNPs among 2 different aggressive cancerous cell lines (K7M2: left; B16-F10: right).

After understanding the drug loading efficiency of the TNPs, we next studied the cellular internalization to evident the targeting capability of TNPs in the *in-vitro* environment against mouse osteosarcoma K7M2 cells. The cellular internalization of TNPs was first visualized under confocal laser scanning microscope by incubating RhB-labeled TNPs with K7M2 cells for 3 h. Confocal micrographs revealed that a lot of NPs internalized into the cell and localized around the periphery into the cellular compartment (Fig. 5A,B) with some localized into the perinuclear region as evident from merged z-stack CLSM images (Fig. 5B).

A quantitative cellular uptake of RhB-labeled TNPs was then conducted using flow cytometry in compared to conventional PEGylated NPs. Our results showed the cellular uptake of both TNPs and PEGylated NPs displayed time-dependent uptake (Fig. 5C). As the nanoparticle incubation time increased, the enhancement of cellular uptake was observed in both TNPs and PEGylated group. However, at an equal incubation time, the TNPs were taken up by K7M2 cells is higher than that of PEGylated NPs (Fig. 5D), suggesting that conjugation with bisphosphonate plays a significant role in targeting nanoparticles to a bone cancer cell in compare to PEGylated NPs.

As most cancer cells display aggressive profile in the unselectively uptake of substances presence in their growing environment, it is important to set up a comparative experiment in order to assess the specificity of targeting ligands among different cancerous cell types. With this in mind, we have chosen another aggressive non-targeted melanoma (B16-F10) cell line to rationally evaluate the distinctive property of TNPs under cellular environment. As a result, a dramatic increase in K7M2 cellular uptake was observed from 30 min to 3 hours of incubation as evidenced by an increase in fluorescence intensity. This time-dependent cellular uptake pattern was absence in the case B16-F10 melanoma cells where fluorescent intensity remains constant even after 3 hours of incubation. The high cellular uptake of targeted nanoparticle toward K7M2 cells was attributed to cell membrane specific interaction with phosphate moiety of Alendronic acid, in a typical endocytosis-mediated receptor uptake. This observation was in agreement with the previous study conducted by T.-K. Ryu *et al*. where Alendronic acid conjugated nanodiamond showed extensive accumulation in osteoblastic cells (MC3T3-E1) but not non-targeted HepG2 and NIH3T3 cell types^27^. Specifically, protein tyrosine phosphatases were investigated as one of the possible bisphosphonate binding receptor presence on the surface of osteoblastic cells^28^. Moreover, protein phosphatases were identified to be overexpressed in osteosarcoma in compared to that of normal osteoblast and osteoclast cells which would help to target ligand to target cancerous cells efficiently^29^,^30^. Also, the same phenomenon was observed by Toledo *et al*. where researchers were studied in the bone tumor of thirty osteosarcoma patients and found that protein tyrosine phosphatases are over expressed in osteosarcoma^30^. Osteosarcoma cells are of the osteoblastic lineage, which is characterized by cells secreting the osteoclast-inducing factor, receptor activator of nuclear factor-κB ligand. Receptor activator of nuclear factor-κB-Fc, osteoprotegerin, bisphosphonates, and Src inhibitor are shown as positive candidates and can control various aspects of osteoclast function^31^. From the results, it could be interpreted that phosphate moiety on the surface of nanoconstruct can selectively facilitate the accumulation of the nanoparticles in the targeted cancerous cells.

### Biocompatibility and cellular cytotoxicity study

**Figure 6:**
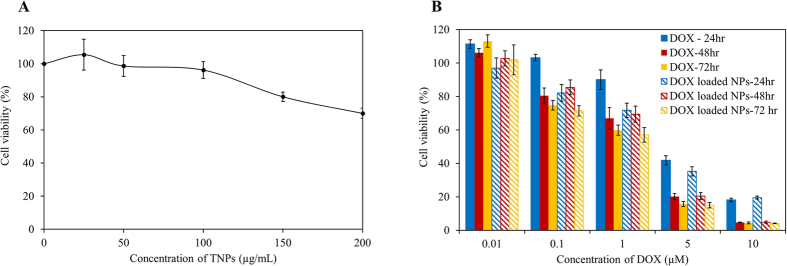
Cellular cytotoxicity studies. (**A**) Concentration dependent cytotoxicity of TNPs. (**B**) Comparative cytotoxicity studies of free Dox and Dox loaded TNPs against K7M2 Osteosarcoma. Values represent mean ± s.d., n = 6.

The biocompatibility of TNPs and *in vitro* cytotoxicity of DOX loaded TNPs were studied in K7M2 Osteosarcoma bone cancer cells using MTT assay. As can be seen in Fig. 6A, at low TNPs concentration, no significant toxicity related to TNPs was observed indicating the excellent biocompatible of ALE-lipid at low concentration. However, when this concentration increases up to 150 ug/mL, cell viability decreases to 80%, this reflection could be explained by masking of the cellular surface under 96-well plate environment, hence reduce the cellular accessibility to oxygen and creating an unfavorable growing environment to cell which further induce unexpected cell death.

In a typical cellular cytotoxicity experiment of free DOX and DOX loaded TNPs, the results revealed that both agents exhibit a time- and dose-dependent cytotoxic effect (Fig. 6B) in which at low incubation time (24 hr) the drug loaded NPs showed higher cytotoxicity than free DOX with the IC50 of 3.7 and 6.1 μM, respectively (Table 1). This enhancement in cytotoxicity of DOX loaded TNPs in lower incubation time is likely due to nanoparticle’s internalization mechanism. First, the exterior phosphate groups of targeted NPs were attracted by protein tyrosine phosphatases receptor present on the surface of osteosarcoma cells leading to the acceleration in accumulation and distribution throughout cell membrane. These targeted nanoparticles further internalized into the cell via endocytosis with the core holding drug; thereby, intensely increase intracellular drug concentration and resulted in enhanced cytotoxicity. Whereas, in the case of free DOX, water soluble drug slowly diffuse into the cell limiting intracellular drug concentration. However, when cells were under treatment for a longer period of time (48 hr and 72 hr), both free DOX and DOX loaded TNPs exert similar cytotoxicity effect. This further supports the evidence of rapid uptake of the nanoparticulate system thereby increasing intracellular drug concentration as compared to that of free drug molecules.

## Conclusion

Targeted therapy holds great potential for minimizing drug related non-specific toxicity. Towards this end, we have demonstrated the target specific delivery of DOX using a nanoparticulate system consisting of the densely packed alendronic acid corona that strongly binds with bone mineral, hydroxyapatite, aiming to target bone microenvironment. Engineered nano-system shows active accumulation into the osteosarcoma cell exhibiting dose-dependent toxicity similar to that of DOX in *in vitro* condition. However, in lower incubation time nanomedicine shows higher toxic effect than that of free DOX, which reveals that the nanomedicine delivers a higher dose of DOX into the cells. Most importantly, higher ionic and serum stability of nanoparticle revealed that decorating nanoparticle surface with alendronic acid provides sufficient hydration layer and strong negative surface charge to sterically stabilized NP, which could be an alternative to PEGylated system to design nanocarrier. Overall, alendronic acid decorated proposed nano-system could provide a promising and most effective platform technology in the treatment of osteosarcoma.

## Materials and Methods

### Chemicals and reagents

Poly(D,L-lactide-co-glycolide) carboxylate end group (50:50, 0.55–0.75 dL/g) was purchased from DURECT Corporation (Birmingham, AL, USA). 1,2-dipalmitoyl-sn-glycero-3-phosphoethanolamine-N-(succinyl) (sodium salt) (16:0 Succinyl PE) and L-α-Phosphatidylethanolamine-N-(lissamine rhodamine B sulfonyl) (Ammonium Salt) (Egg-Transphosphatidylated, Chicken) (Egg Liss Rhod PE) were purchased from Avanti Polar Lipid Inc. (Alabaster, AL, USA). Alendronic acid was purchased from TCI America. 1-ethyl-3-(3-dimethylaminopropyl)carbodiimide hydrochloride (EDC) and N-hydroxysuccinimide (NHS) were purchased from Sigma-Aldrich (Milwaukee, WI, USA). Doxorubicin hydrochloride salt was purchased from LC Laboratories (Woburn, MA, USA). Osteosarcoma Cell line K7M2 and Mouse Melanoma (B16-F10) were purchased from ATCC and maintained according to the manufacturer’s recommendation. All other chemicals and solvents were purchased from Sigma-Aldrich (Milwaukee, WI, USA) and used as received.

### Synthesis of lipid bisphosphonate conjugates

Alendronic acid conjugated lipid (ALE-lipid) was synthesized by simple coupling chemistry initiated by EDC and NHS. Briefly, 15 mg of Succinyl PE lipid was dispersed in 2-(*N*-morpholino)ethanesulfonic acid (MES) buffer (4 mL, pH = 4.5), activated by 35.9 mg EDC and 37.5 mg of NHS and stirred at room temperature for 15 min. To this 47.4 mg Alendronic acid (ALE) in 6 mL Phosphate Buffered Saline (PBS) containing 10% triethylamine (TEA) was added and stirred for additional 24 h at room temperature. In order to purify the product, conjugated lipid was placed inside the benzoylated cellulose dialysis bag (MWCO ~500 Da), and dialyzed against water for 24 hours at room temperature. The samples were lyophilized to obtain a dry powder and stored at −20 °C for further use. FT-IR and ^1^H-NMR were used to confirm the formation of ALE-lipid.

### Nanofabrication of lipid bisphosphonate nanoparticles and multifunctional polymeric nanoparticles

Targeted hybrid nanoparticles were prepared by single step nanoprecipitation. In brief, 400 μL of PLGA (1 mg) in acetonitrile was added dropwise to 2 mL of 200 μg ALE-lipid (dispersed in 4% ethanol) under a magnetic stirring condition at 60 °C. To this, 1 mL of Mili-Q water was added to cool down the mixture and stirred continuously for additional 1 hour to facilitate the formation of nanoparticles and evaporation of organic solvent at room temperature. The TNPs were further purified using Amicon Ultra-4 centrifugal filter (Millipore, MA) with a molecular weight cut-off of 10 kDA and stored at 4 °C for further use.

For DOX-loaded TNP preparation, different amount of DOX (10, 25, 50, 100, and 150 μg) were mixed with 100 μL of PLGA (10 mg/mL) and dried under vacuum. A polymeric film with the drug was then dissolved in 400 μL acetonitrile prior to nanoparticle preparation. Controlled PLGA nanoparticles were also prepared by dropwise adding 100 μL of PLGA (1 mg) in acetonitrile to 1 mL of Milli-Q water and purified following aforementioned protocol.

Rhodamine dye labelled TNPs were also prepared by hydrating 20 μg of L-α-Phosphatidylethanolamine-N-(lissamine rhodamine B sulfonyl) (Ammonium Salt) (Egg Liss Rhod PE) film with 200 μL ALE-lipid (1 mg/mL) before performing nanoprecipitation process.

RhB-labeled PEGylated nanoparticles were prepared by adding 400 μL PLGA (1 mg) into 2 mL 4% ethanol containing 200 μg DSPE-PEG-suc, 260 μg DSPG, and 20 μg of L-α-Phosphatidylethanolamine-N-(lissamine rhodamine B sulfonyl) (Ammonium Salt) (Egg Liss Rhod PE) under magnetic stirring condition at 60 °C, followed by addition of 1 mL Milli-Q water and purified as described above.

### Characterization of nanoparticles

The hydrodynamic size and zeta potential measurements of the prepared TNPs and DOX-loaded TNPs were analyzed by Dynamic light scattering (DLS) using a Zetasizer Nano ZSP (Malvern, Worcestershire, UK). The Smoluchowski model was used to calculate the zeta potential value. All data represents the average of triplicate measurements of samples prepared in different preparations. The morphology of the prepared TNPs was further analyzed using Transmission Electron Microscope (TEM, Tecnia G2, Spirit Bio TWIN). TEM samples were prepared by drop casting and evaporation technique using formvar coated copper grid (400 mesh). TEM images were analyzed by GATAN digital imaging system (GATAN, Inc.). The amount of encapsulated DOX and the resulting encapsulation efficiency was quantified spectrophotometrically using UV-VIS microplate reader by measuring the absorbance at 490 nm.

### Stability study of nanoformulation

The stability of TNPs in physiological ionic condition was investigated at pH 7.4 using PBS. In brief, 500 μL of 1 mg/mL nanoparticles suspension were added to 500 μL of 2X PBS and incubated at 37 °C with a rotating motion for 7 days. The stability of nanoparticles was determined by measuring the particle size and PDI every 24 hr. The serum stability of the prepared PLGA NPs and TNPs were carried out as reported^26^,^32^,^33^. Specifically, 100 μL of 1 mg/mL nanoparticles were incubated with 100 μL of 10% Fetal Bovine Serum at 37 °C and measure its change in absorbance at 560 nm kinetically every 5 s over a period of 1 h, double orbital shaking with slow speed was applied prior to each measurement using Microplate reader (BioTek, Synergy H1 hybrid reader).

### Calcium binding affinity

Nanoparticles engineered herein are highly monodispersed and recovered as an aqueous suspension. Under mild centrifugation (1000 to 3000 rpm for 10 mins), they are not pelleted down. We have taken this advantages for Ca^2+^ binding assay. The binding affinity of TNPs to Calcium was determined indirectly by measuring the fluorescence intensity of RhB-labelled NPs presence in the supernatant and compare to the initial fluorescence intensity of NPs fluorescent spectrophotometrically. To optimize binding assays, we first used a different concentration of RhB-labelled TNPs (100, 200, 400, 600, 800, and 1000 μg/mL) to incubate with 5 mg Hydroxyapatite (HAp) for 30 mins at 37 °C. At the end of incubation time, samples were centrifuged at 1,500 rpm for 5 min to spin down HAp aggregates and the nanoparticles that bound to them. 100 μL of supernatant was used to indirectly quantify the relative amount of nanoparticles bind to HAp. In the case of kinetic binding experiment, 1 mL of RhB-labelled TNPs (100 μg/mL and 1 mg/mL) were incubated with 5 mg HAp in macro-crystal form for varying periods of time (30 s, 2, 5, 15, 30, 60, 120, and 240 mins) at 37 °C and processed as aforementioned protocol. In addition, RhB-labelled PEGylated NPs (100 μg/mL and 1 mg/mL) were used as controlled particles followed the same experimental condition.

### Drug release study

The cumulative drug release from the DOX loaded TNPs was assessed under a physiological condition at 37 °C. In brief, DOX loaded TNPs (25 μg/mL, 1 mL) were placed in a dialysis bag membrane (Mw. Cutoff = 10 kDa) and dialyzed against 250 mL of PBS (pH = 7.4). At constant stirring (100 rpm), 200 μL of the sample was taken at predetermined time intervals. The amount of released DOX was quantified by measuring the DOX fluorescence with the excitation and emission wavelength of 490 nm and 580 nm, respectively. As a control experiment, 25 μg/mL of Free DOX was placed in a dialysis bag and processed under the same condition.

### Intracellular uptake study

In order to verify cellular uptake efficiency of ALE-Lipid decorated NPs, RhB-labeled TNPs was used in mouse Osteosarcoma bone cancer cell line (K7M2). In brief, cells were seeded in Poly-D-lysine coated 8 chamber slide at a density of 50,000 cells per well and incubated for 24 h. Then, the cells were treated with 50 μg/mL RhB-labeled TNPs suspension prepared in complete DMEM and incubated for 4 h. After incubation, treated cells were washed twice with 1X PBS (pH 7.4), fixed with 4% paraformaldehyde for 30 min at room temperature, stained with DAPI for additional 10 min and imaged under a Confocal Laser Scanning Microscope (Carl Ziess, LSM-700).

### Fluorescence-activated cell sorting (FACs) studies

To quantitatively evaluate the cellular internalization efficiency of ALE-Lipid decorated NPs, a comparative experiment was conducted on targeted Osteosarcoma cell line and non-targeted Melanoma (B16-F10) cells. In brief, K7M2 cells (or B16 cells) were seeded in T25 tissue culture flasks at 4 × 10^6^ cells per flask for 24 hr. After incubation, cells treated with 2 mg RhB-labeled TNPs suspended in DMEM. Cells were then incubated at 37 °C at varying periods of time (30 min, 1, and 3 h) at 37 °C. After incubation, cells were washed twice with ice-cold PBS, detached with 0.08% w/v trypsin and analyzed on a flow cytometer. RhB-labeled PEGylated NPs at the same concentration were used as control particles.

### *In vitro* cytotoxicity assays

The *in-vitro* cytotoxicity of TNPs was conducted on Osteosarcoma K7M2 using MTT assay. In brief, 2 × 10^4^ cells per well in DMEM medium were seeded in a 96-well plate and incubated for 24 h. After incubation, the media were replaced with different TNPs concentration (10, 25, 50, 100, 150 and 200 μg/mL) and DOX loaded TNPs along with free DOX (0.01, 0.05, 0.1, 0.5, 1, 2, 3 and 5 μM) and incubated for additional 24, 48, and 72 hr. Control cells were also maintained without any TNPs treatment (n = 6). After incubation, MTT was added to each well and further incubated for 3 h according to the manufacturer recommendation. The insoluble formazan crystals were solubilized using DMSO and their absorbance was recorded at 570 nm using a microplate reader (BioTek, Synergy H1 hybrid reader).

## Additional Information

**How to cite this article**: Nguyen, T. D. T. *et al*. Engineered Nanomedicine with Alendronic Acid Corona Improves Targeting to Osteosarcoma. *Sci. Rep.* **6**, 36707; doi: 10.1038/srep36707 (2016).

**Publisher’s note:** Springer Nature remains neutral with regard to jurisdictional claims in published maps and institutional affiliations.

## Figures and Tables

**Table 1:** IC50 of free DOX and DOX loaded TNPs on K7M2 cells.

Incubation time	IC50 value (μM)	
	Free DOX	DOX loaded NPs
24 hr	6.144	3.793
48 hr	2.621	3.064
72 hr	1.44	1.717
